# Professional Dualities

**Published:** 1920-05-22

**Authors:** Sidney Blow

**Affiliations:** Turville Heath, Henley-on-Thames.


*To the Editor of* THE HOSPITAL.

### "Professional Dualities."

*To the Editor of* THE HOSPITAL.

SIR,—Our village nurse is a reader of THE HOSPITAL, and has drawn my attention to the enclosed [May 8, p. 134], which is quite incorrect. Dr. Maurice Nicoll and his sister wrote a novel entitled "Lord Richard in the Pantry." And as Mr. Douglas Hoare and myself had written a similar story in play form before discovering that "Martyn Swayne" had had a similar idea. So we incorporated part of the dialogue of Lord Richard—namely, in Act. I.—and acknowledged them on the programmes as authors of the *Novel*.

The play and the novel are very very different, so perhaps you would be so kind and correct the statement in your paper of May 8, as it is misleading.—Yours truly,

SIDNEY BLOW.

Turville Heath, Henley-on-Thames.

[Mr. Blow's letter makes clear the point he wishes to establish. While expressing regret that our attribution of the authorship of the play, "Lord Richard in the Pantry" to Dr. Nicoll should have fallen short in any way of the full truth, there is this much to add, that the hoardings of London carry at this moment advertisements of the play subscribed "Adapted from the novel by Martin Swayne" (the pseudonym of Dr. Nicoll and his sister).—ED., THE HOSPITAL.]

